# Mechanisms That Enhance Sustainability of p53 Pulses

**DOI:** 10.1371/journal.pone.0065242

**Published:** 2013-06-03

**Authors:** Jae Kyoung Kim, Trachette L. Jackson

**Affiliations:** Department of Mathematics, University of Michigan, Ann Arbor, Michigan, United States of America; University of Chicago, United States of America

## Abstract

The tumor suppressor p53 protein shows various dynamic responses depending on the types and extent of cellular stresses. In particular, in response to DNA damage induced by γ-irradiation, cells generate a series of p53 pulses. Recent research has shown the importance of sustaining repeated p53 pulses for recovery from DNA damage. However, far too little attention has been paid to understanding how cells can sustain p53 pulses given the complexities of genetic heterogeneity and intrinsic noise. Here, we explore potential molecular mechanisms that enhance the sustainability of p53 pulses by developing a new mathematical model of the p53 regulatory system. This model can reproduce many experimental results that describe the dynamics of p53 pulses. By simulating the model both deterministically and stochastically, we found three potential mechanisms that improve the sustainability of p53 pulses: 1) the recently identified positive feedback loop between p53 and Rorα allows cells to sustain p53 pulses with high amplitude over a wide range of conditions, 2) intrinsic noise can often prevent the dampening of p53 pulses even after mutations, and 3) coupling of p53 pulses in neighboring cells via cytochrome-c significantly reduces the chance of failure in sustaining p53 pulses in the presence of heterogeneity among cells. Finally, in light of these results, we propose testable experiments that can reveal important mechanisms underlying p53 dynamics.

## Introduction

p53 protein is one of the most important tumor suppressors that is mutated in more than half of all human cancers [1]. The p53 protein regulates key functions, such as DNA repair, cell cycle arrest and apoptosis, which prevent tumorigenesis in response to cellular stress (e.g. DNA damage and genomic instability) [2]. Recent studies have found that depending on the type of stresses, p53 protein exhibits different dynamical behaviors. While p53 protein levels are low in the absence of stress, transient DNA double-strand breaks (DSBs) that occur during a normal cell-cycle lead to spontaneous pulses of p53 [3]. A single pulse of p53 can also be triggered by UV irradiation [4]. The most dynamic behavior of p53 is induced by severe DNA damage. When severe DSBs are caused by γ-irradiation or radiomimetic drugs, cells generate a series of p53 pulses [5], [6], [7], [8]. Interestingly, these different dynamical behaviors appear to be highly correlated with appropriate responses of p53 to different types of stresses [3], [4], [9].

The γ-triggered p53 pulses have three notable features: 1) the pulse amplitudes are independent of γ-irradiation strength, 2) the pulses are sustained so long as DSBs persist (i.e. undamped pulses) and 3) while the period of the pulses is tightly regulated, the amplitude is highly variable. The molecular mechanisms underlying these unique features of p53 pulses have been explored both experimentally and theoretically. Among many feedback loops regulating p53 [10], a negative feedback loop between p53 protein and E3 ubiquitin ligase Mdm2 is considered to be a core mechanism that generates p53 oscillations [11], [12], [13], [14], [15], [16]. Recently, mathematical modeling and subsequent experiments have found an additional feedback loop between upstream kinase ATM and p53 through WIP1, which is also required to sustain p53 pulses with amplitudes that are independent of γ-irradiation strength [5], [17]. In addition to the molecular mechanisms underlying p53 pulses, the relationship between the dynamics of p53 and its output functions, such as DNA repair or cell cycle arrest have also been widely studied both theoretically and experimentally [3], [9], [18], [19], [20], [21], [22]. In light of the considerable theoretical and experimental focus on dynamics of p53 pulses and the role of these pulses, it is somewhat surprising that very little attention has been paid to how cells robustly sustain p53 pulses, even in the presence of perturbation (e.g. intrinsic noise or genetic perturbation) [14], [22]. Given the importance of sustaining p53 pulses for cell fates in response to severe DNA damage [3], [9], it is of considerable interest to understand how cells can sustain p53 pulses over a wide range of conditions.

In other biological oscillatory systems, mechanisms that sustain robust rhythms in the presence of perturbations have been widely studied. Importantly, it has been shown that adding additional positive or negative feedback loops to a core negative feedback loop can often contribute to maintaining rhythms over a wide range of environmental conditions [23], [24], [25]. For instance, an additional positive feedback is essential for high amplitude rhythms of active mitosis promoting factor (MPF) in the presence of intrinsic noise [24], [26]. An additional negative feedback loop also allows molecular circadian rhythms to persist in the presence of genetic perturbations [23]. Together with additional *intracellular* feedback loops, *intercellular* feedback loops through coupling among neighboring cells also often contribute to generating robust rhythms. For instance, while the circadian rhythms of clock gene expression in single cells are easily disrupted by intrinsic noise or genetic mutations, circadian rhythms in coupled cells can persist robustly even in the presence of significant genetic perturbations [27], [28]. Cellular coupling can also tightly regulate the periods and the amplitudes of c-AMP rhythms in *Dictyostelium* [29] and membrane potential spikes in neurons [30], . The coupling has been investigated as a mean to reduce the effects of noise on rhythms, but more recent studies have found that coupling and noise often synergistically enhance rhythms in calcium systems and circadian clocks [28], [32].

The identification of mechanisms that enhance robustness of rhythms in various biological oscillatory systems has lead to the question of whether similar mechanisms that enhance robust γ-triggered p53 pulses exist. Indeed, several positive feedback loops acting on p53 through PTEN, dapk1, c-Ha-Ras and DDR1 have been identified [33], [34], [35], [36]. However, these feedback loops do not appear to be essential for sustaining p53 pulses [37]. A recent study identified a novel positive feedback loop between p53 and Rorα, which may enhance the sustainability of p53 pulses [38]. In this positive feedback loop, p53 promotes gene expression of Rorα in response to DNA damage, and increased RORα protein stabilizes p53. Together with additional feedback loops acting on p53, a recent study found radiation induced bystander effect (RIBE) [39], which may be a potential mechanism that couples p53 pulses of neighboring cells. RIBE refers to DNA damage of *un-irradiated* cells induced by the molecular signals produced by their neighboring *irradiated* cells. Among various molecular signals involving RIBE, Cytochrome-c (cyt-c) can act as an excitatory signal for p53 pulses in neighboring cells [40]. More specifically, p53 activation induced by DNA damage stimulates the mitochondrial release of cyt-c, which activates p53 in *un-irradiated* neighboring cells. However, whether cyt-c can couple p53 pulses in *irradiated* cells has yet to be investigated.

Here, we develop a new mathematical model of p53 to explore molecular mechanisms that enhance sustainability of p53 pulses in the presence of DNA damage. Our new model is able to reproduce many key experimental observations that discern characteristics of p53 pulses. By simulating this model both deterministically and stochastically, we find that a positive feedback loop between p53 and Rorα plays a pivotal role in sustaining p53 pulses over a wide range of conditions. Moreover, we find that noise can often prevent p53 pulses from dampening even after mutations of key molecular species that generate p53 pulses. Interestingly, we also found that p53 pulses in DNA-damaged cells have characteristics similar to Type II resonator neurons, which are prone to synchronize their spikes through excitatory couplings. Similar to Type II neurons, even a weak coupling via cyt-c can synchronize p53 pulses of cells and significantly increase the chance that p53 pulses will be sustained in response to DNA damage.

## Results

### Model Description

**Figure 1:**
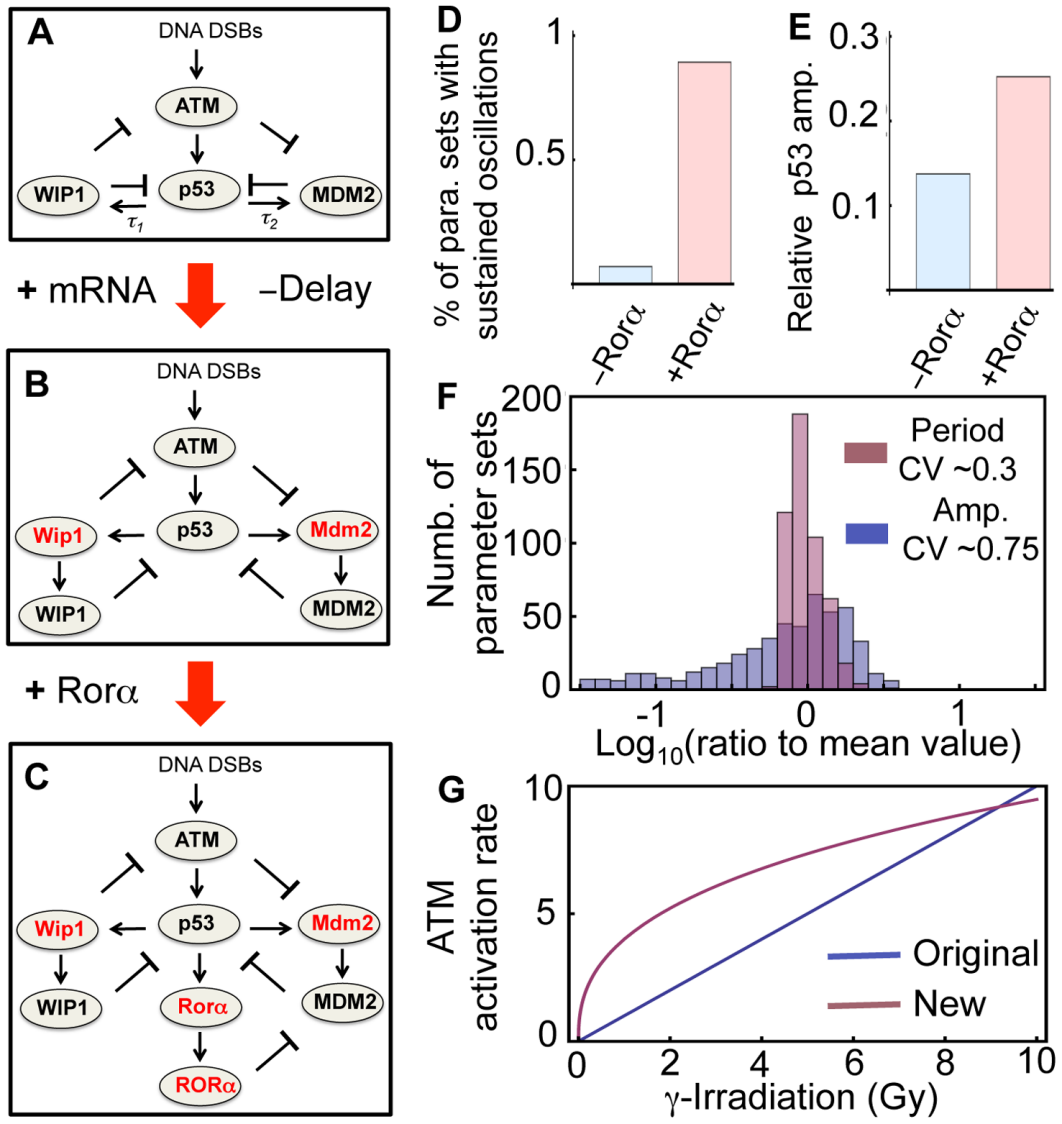
The new model of p53 dynamics. **A**. A schematic diagram of Batchelor’s DDE model [5]. **B**. Extension of Batchelor’s model that includes intermediate steps instead of explicit time delays. **C**. Our full model including an additional positive feedback loop between p53 and Rorα as well as intermediate steps (see Materials and Methods and Text S1 for a detailed model description). **D–E**. Comparison between the model without Rorα (Figure 1B) and with Rorα (Figure 1C) indicating the probability of p53 rhythm occurrence (**D**) and average relative amplitude of p53 (**E**). For models without and with Rorα, newly added parameters were randomly chosen until 500 parameter sets were found that generate sustained pulses of p53 in response to 10 Gy irradiation. For 500 parameter sets, mean relative amplitude of p53 pulses were measured in the two different models. **F**. The distributions of periods and amplitudes of our new model (Figure 1C) with the 500 parameter sets. **G**. In the Batchelor’s model, the activation rate of ATM is proportional to the strength of γ-irradiation [5], but in the new model, the activation rate of ATM saturates for the strong γ-irradiation.

To explore mechanisms that enhance sustainability of p53 pulses, we have developed a new mathematical model that describes the dynamics of p53 pulses. Our model is based on the delay-differential equation (DDE) model of Batchelor et al. 2008, which made important contributions to understanding the mechanisms underlying p53 pulse generation [3], [4], [5], [9]. In particular, their model found that DNA damage induced activation of ATM, an upstream signaling kinase for p53 phosphorylation, and that deactivation of ATM by p53 via WIP1 triggers a series of p53 pulses [5]. Batchelor’s model tracks the temporal changes of five molecular species: p53_inactive_, p53_active_, Mdm2, Wip1 and ATM-P (Figure 1A). The interactions among these five species can be described by three negative feedback loops:

1. ATM-P induced p53_active_ promotes production of Mdm2 and Mdm2 induces ubiquitination of p53,
2. ATM-P induced p53_active_ promotes production of Wip1 and Wip1 mediates dephosphorylation and inactivation of p53_active_, and
3. ATM-P induced p53_active_ promotes the production of Wip1 and Wip1 dephosphorylates and inactivates ATM-P [4], [5].

In addition to these negative feedback loops, Mdm2 phosphorylation by ATM-P inactivates and destabilizes Mdm2.

We begin developing our new model by first converting the DDEs used in Batchelor’s model into ordinary differential equations (ODEs). Our rationale for this modification is that DDEs often generate rhythms in systems whose structures are not likely to produce rhythms naturally [25], [41]. We, therefore, want to ensure that the p53 pulses in our model are not explicit delay-induced instabilities, which often occur in nonlinear feedback systems. Another reason for utilizing ODEs is that it is more difficult to perform stochastic simulations of DDEs than ODEs because methods to introduce stochasticity into the explicit time delays in DDEs have not been fully developed [42]. Two explicit time delays, 0.7 hours and 1.25 hours for p53_active_ -dependent production of Mdm2 and Wip1, respectfully were used in Batchelor’s model. We removed these explicit delays and introduced intermediate steps (mRNAs) required for the production of MDM2 protein and WIP1 protein (Figure 1B and Materials and Methods). To test whether the newly introduced intermediate steps without the explicit time delays are sufficient to generate sustained p53 pulses after 10 Gy irradiation, new parameters associated with the equations for the mRNA of Mdm2 and Wip1 were randomly searched until 500 parameters yielding oscillations were found (see Methods and Materials for details of parameter searching). Here, we defined sustained pulses as undamped oscillations (see Methods and Materials for details). However, the probability of rhythm occurrence was very low (<0.1%) (Figure 1D). Considering robustness as a design principle of biological systems whose essential functions are nearly independent of varying biochemical parameters [43], [44], [45], the low chance of rhythm occurrence implies that the current ODE model with intermediate steps for protein production might lack one or more essential components for generating rhythms.

One potential issue with the current ODE model is that it consists of only negative feedback loops. It is well known that an additional positive feedback loop often enhances the robustness of rhythms in other biological oscillatory systems [14], [24], [25]. Indeed, a recent study identified a positive feedback loop between Rorα and p53, in which after γ-irradiation, p53_active_ induces the expression of Rorα and increased RORα protein inhibits the Mdm2 dependent degradation of p53 [38], [46]. After adding this positive feedback loop into the model (Figure 1C and Materials and Methods), we randomly searched the new parameters associated with this positive feedback loop as well as those for the mRNA of Mdm2 and Wip1 again. Surprisingly, the addition of this positive feedback loop significantly increased the chance of rhythm occurrence (>10 fold) (Figure 1D). Another benefit of adding the positive feedback loop is that it increases the average amplitude of sustained p53 pulses generated with the 500 parameter sets (Figure 1E). We also analyzed the distributions of periods and amplitudes of pulses induced by these 500 parameter sets (Figure 1F). With the additional positive feedback loop, the new ODE model was able to capture a distinct characteristic of p53 pulses: a large variation in amplitude, but little variation in periods [7]. Since recent studies have shown that the sustainability of p53 pulses is essential for the repair of DNA damage, our study further indicates that the positive feedback loop between p53 and Rorα can play an important role in the appropriate response of p53 to DNA damage [9]. Indeed, Rorα^−/−^ cells failed to regulate apoptosis in response to DNA damage [38].

Finally, previous experimental studies found a nonlinear relationship between the amount of DNA damage and the activation rate of ATM [47], [48]. We therefore changed the mechanisms for ATM activation induced by DSBs in Bachelor’s model, where ATM activation is linearly proportional to the extent of DNA damage [5]. In the new model, ATM activation becomes more sensitive to small amounts of DNA damage and becomes saturated for large amounts of DNA damage (Figure 1G and Materials and Methods). We found that this modification is critical to simulating the correct responses of p53 systems, such as period distributions in response to different strengths of γ-irradiation (see below).

### Model Validation

**Figure 2:**
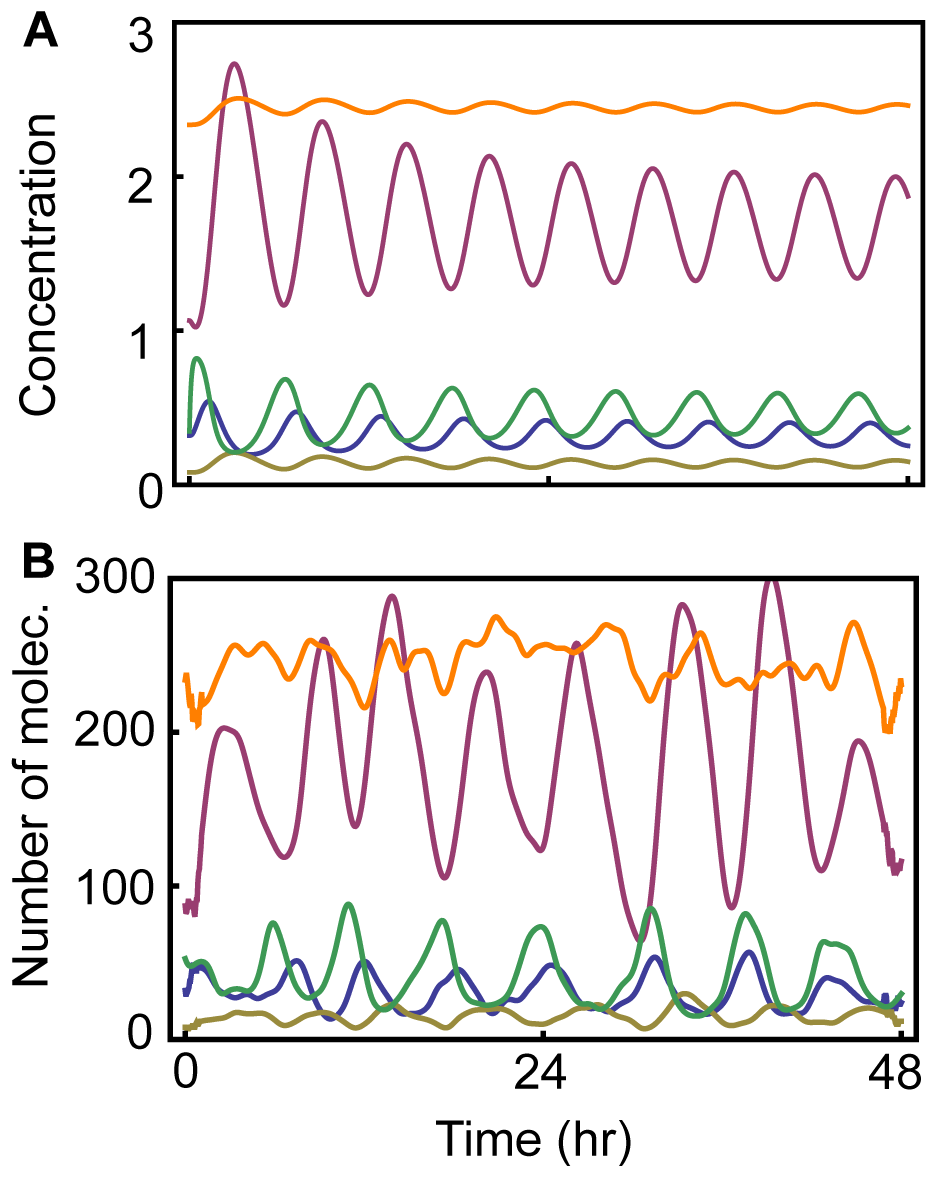
Deterministic and stochastic simulation of the new model after 5 Gy irradiation. **A**. Deterministic Simulation. **B**. Stochastic simulation with Gillespie algorithm. Total p53 (Blue), active ATM (Green), WIP1 (Yellow), MDM2 (Brown), and RORα (Orange).

Our extensions and modifications of Batchelor’s model added 14 parameters to the list of parameters in their model. We selected new parameters that were able to reproduce many important experimental findings, including the mean and variation of the period and amplitude of p53 pulses with various strengths of the irradiation dose and mutation phenotypes (Table S1). Other parameter values were maintained from the original model, which were carefully selected based on experimental data [5]. We also analyzed the sensitivity of all parameters (Table S1 and Figure S1) and found that all species in the model play important role in regulating p53 pulses. With the new parameter set, our model was able to generate sustained pulses of p53 in response to 5 Gy irradiation (Figure 2A). To compare simulations of the new model with experimental data, stochastic simulations were also performed with Gillespie’s algorithm to reflect molecular fluctuations in experimental data [49]. Gillespie’s algorithm has been widely used to study the effect of molecular noise on the dynamics of biochemical and genetic systems [11], [50], [51]. The lists of reactions together with the probability for their occurrence are described in Table S2. Hill equations and Michaelis-Menten equations of the deterministic model were also used in the stochastic simulation by assuming fast time scales for the elementary reactions underlying these equations [52], [53]. This stochastic model also generated sustained p53 pulses after 5 Gy irradiation (Figure 2B).

**Figure 3:**
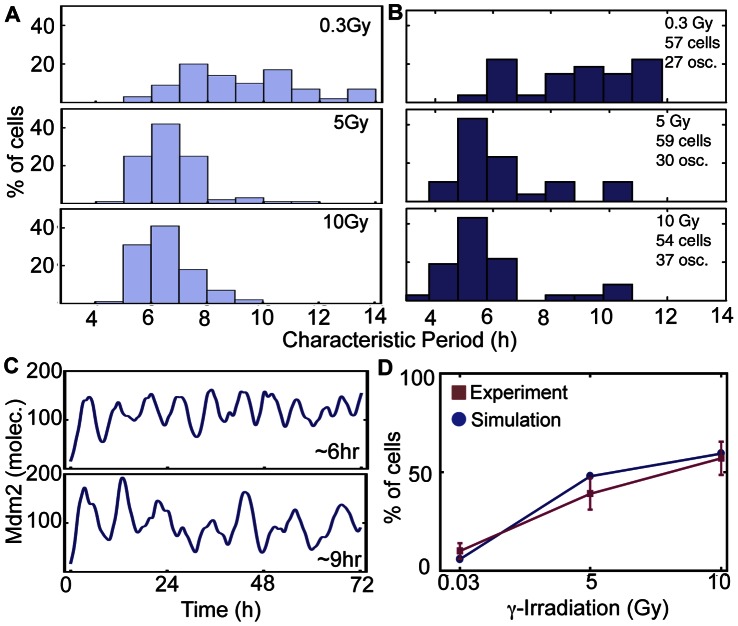
Period distributions of cells in response to different strengths of γ-irradiation. **A**. Simulated histograms of the characteristic period of Mdm2 in 100 cells over the first 72 h after irradiation in response to different strengths of irradiation (see Materials and Methods for the details of period detection method). **B**. Experimentally measured histograms of the characteristic period of Mdm2-YFP signals in MCF-7 cells exposed to different strengths of γ-irradiation (Fig. 3 in [7]). Reprinted by permission from Macmillan Publishers Ltd: Molecular Systems Biology 2006 (doi: 10.1038/msb4100068). **C.** Examples of simulated Mdm2 oscillations with short (∼6 hr) and long (∼9 hr) periods after 0.3 Gy irradiation. **D**. Fractions of cells (out of the total number of cells) with a characteristic period of 4–7 h in response to different strengths of irradiation match experimental data [7].

**Figure 4:**
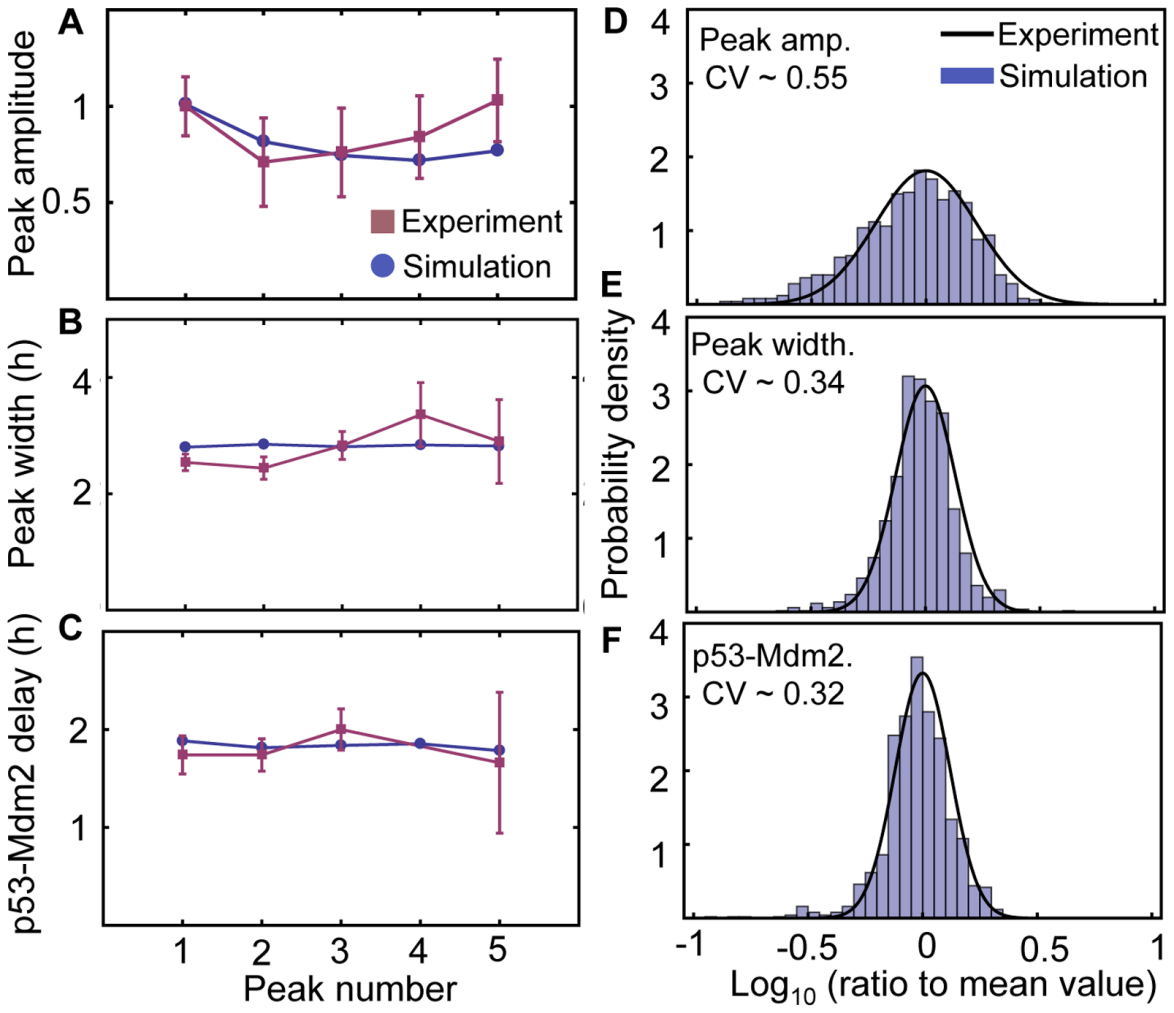
Average amplitude, width, and time delay of oscillation peaks and their variance. **A–C**. Stochastically simulated average values of first five p53 pulse peaks in 200 cells exposed to 5 Gy irradiation match experimental data (Fig. 4 in [7]). **A.** Average amplitude. **B**. Average peak width (full width with half-amplitude). **C**. Average time delay between the p53 peak and the subsequent Mdm2 peak. **D–F.** Simulated distributions of the individual peak amplitudes, peak widths, and time delays between the p53 peaks and Mdm2 peaks divided by its mean value match those measured experimentally (Fig. 4 in [7]). In particular, while amplitude shows a large variation, but peak width and peak delay show small variations. Experimental data is reproduced by permission from Macmillan Publishers Ltd: Molecular Systems Biology 2006 (doi: 10.1038/msb4100068).

First, with Gillespie’s algorithm, we simulated the period distributions of MDM2 oscillations in 500 cells after 0.3, 5, and 10 Gy irradiations. To consider the heterogeneity among cells [54], the original parameters of the model were randomly perturbed by multiplying *e^X^* (*X ∼ N*(0, 0.2)). The simulations showed that the distributions of periods become narrower as γ-irradiation strength increases, which is consistent with experimental data (Figure 3A and 3B) [7]. With low strength γ-irradiation (0.3 Gy), MDM2 rhythms showed a wide range of periods (e.g. 9 hr and 6 hr) (Figure 3C). Moreover, as the strength of γ-irradiation becomes stronger, the fraction of cells oscillating with period 4–7 hours increases, matching experimental data (Figure 3D) [7]. Next, among oscillating cells, we measured the average amplitude, peak width of p53 and time delay between the peaks of p53 and Mdm2 for first five peaks of pulses (Figure 4A–C). All of these values were well matched with experimental data [7]. Moreover, the model simulated a significantly larger variation in the peak amplitudes than those in the peak width of p53 or delay between peak timing of p53 and Mdm2 (Figure 4D–F), as shown in previous experiments [7]. Taken together, the new model can successfully reproduce key features of p53 pulses induced by γ-irradiation.

### Noise can Enhance the Sustainability of p53 Pulses in Mutated Cells

**Figure 5:**
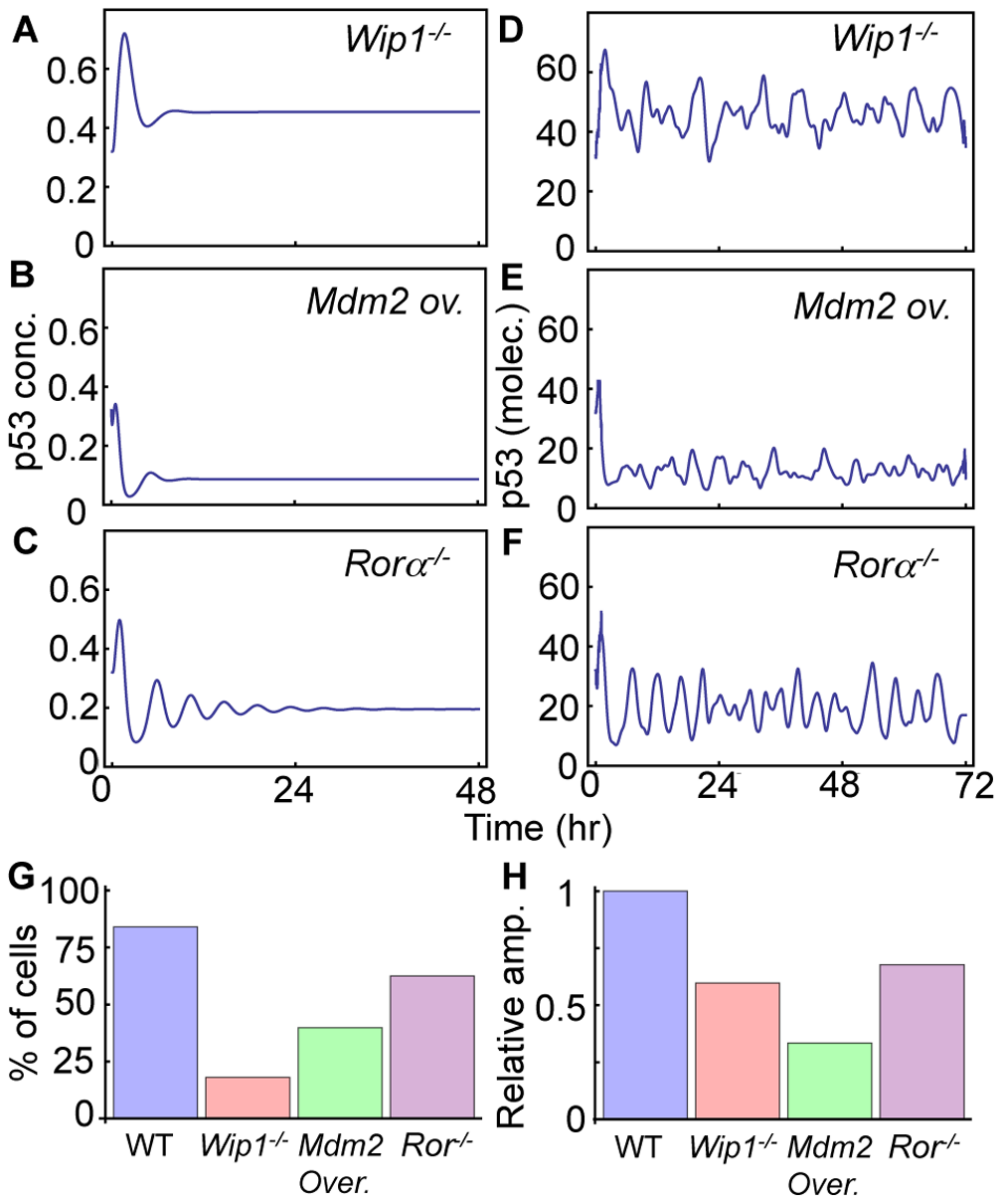
Simulations of knockout or overexpression mutations. **A–C**. Deterministic simulations of *Wip1^−/−^*, *Mdm2* overexpression (10 fold), and *Rorα^−/−^* showed dampened p53 pulses after 10 Gy irradiation. **D–F**. Stochasticity or intrinsic noise was often able to induce sustained p53 pulses even with these mutations after 10 Gy irradiation. **G**. Probability of sustained p53 pulse occurrence with noise for 500 cells in response to 10 Gy irradiation. See Materials and Methods for details regarding the method used to determine whether simulated timecourses are oscillating. **H**. Mean relative amplitude of p53 pulses of oscillating cells among 500 cells (normalized to average relative amplitudes in WT cells).

To quantify the role of each molecular species in the model, we simulated various types of mutations. Given 10 Gy irradiation, the model with Wip1 knockout generates a single peak followed by a high steady state of p53 instead of sustained pulses of p53, matching a previous experimental study (Figure 5A) [5]. It is known that SNP309 (polymorphism of mdm2 promoter) increases expression of Mdm2 mRNA about 10 times and induces the low stable state of p53 instead of oscillations after γ-irradiation [55], [56]. We tested this in our model by increasing the transcription rate of Mdm2 by a factor of 10 and found that this causes the same behavior that was observed experimentally (Figure 5B). Finally, the model predicts that Rorα is also essential to generate sustained pulses of p53 in the presence of 10 Gy γ-irradiation (Figure 5C).

While deterministic simulations predict that sustained p53 pulses will not occur with these three types of mutations, stochastic simulations predict that the sustained pulses often occur even with these mutations (Figure 5D–F). In particular, the model showed clear oscillations of p53 in Rorα^−/−^ cells. Interestingly, previous studies also showed that noise could often induce rhythms in other biological oscillatory systems, such as the circadian clock, by moving the system away from its natural steady state [28]. To see how often noise can induce the sustained pulses with these mutations, we conducted a simulation of 200 cells with Gillespie algorithm. Here, we again assumed heterogeneity among cells as we did previously. Cells with any one of three mutations showed a lower chance of rhythm occurrence than WT cells (Figure 5G). In particular, Wip1^−/−^ cells had the lowest probability of sustaining pulses. Moreover, the amplitudes of pulses were also significantly reduced with these mutations (Figure 5H). Interestingly, a previous experimental study also showed that only small portion of cells can generate sustained p53 pulses with low amplitudes after treatment of Wip1 siRNA [5]. Our simulations indicate that while these mutated cells were unable to generate sustained pulses of p53 after γ-irradiation at the cell population level (deterministic simulation), noise can often induce sustained rhythms at the single cell level (stochastic simulation).

### Common Characteristics between the p53 Model and Type II Neurons

We noticed that the p53 regulatory system in cells behaves like an excitatory system similar to neurons. That is, while p53 levels remain at a steady state in the absence of DNA damage, transient DNA damage can induce spontaneous pulses of p53 [3]. Moreover, severe DNA damage induced by γ-irradiation yields a series of p53 pulses with a small variation in period, but a large variation in amplitude (Figure 4D–E) [7]. Interestingly, these are also common features of type II neurons, which are also known as resonator neurons due to their preferred frequency of spiking [57]. When an external current greater than a certain threshold is applied, type II neurons begin generating spikes with a narrow range of frequencies, regardless of the strength of the external stimuli, while other types of neurons (e.g. type I neurons) yield spikes with a wide range of frequencies depending on the strength of the external currents [57], [58], [59].

**Figure 6:**
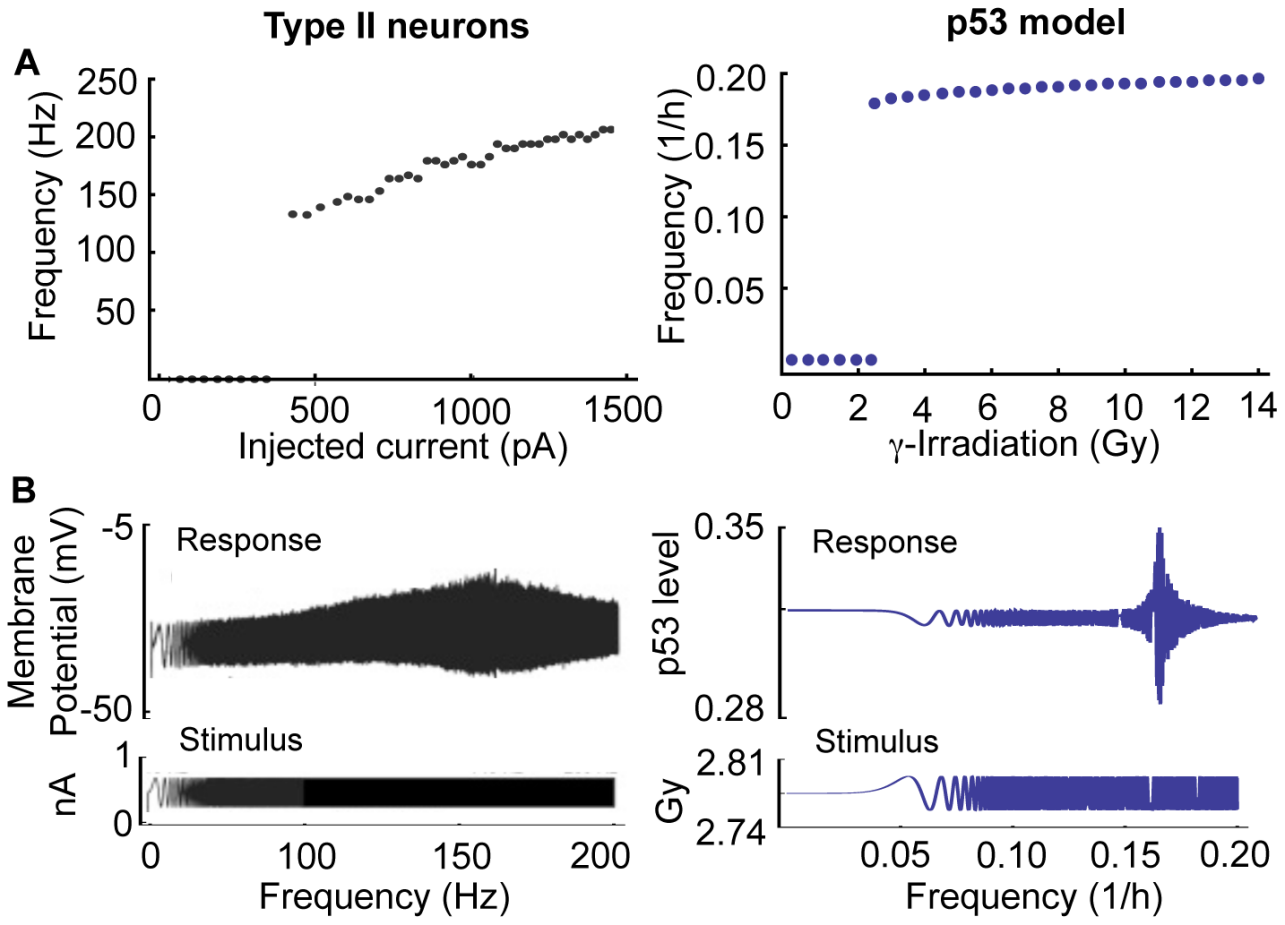
p53 pulses in the model and spikes of type II neurons show similar responses to the external stimuli with different strengths (A) and with varying frequencies (B). **A**. Both oscillating spikes in type II neurons and sustained p53 pulses of model have a non-zero lower bound for frequencies and a narrow range of frequencies in response to different strengths of constant external stimuli. **B**. Both membrane potential in type II neurons and p53 in the model respond sensitively to oscillating stimuli with a specific frequency. The figures of type II neurons are reproduced with Figures 7.3 and 7.17 in [73] by permission from The MIT Press.

We further explored whether pulses of p53 in cells and spikes of type II neurons have more common features in response to external stimuli. Given external constant currents with different strengths, type II neurons have a non-zero lower bound for frequency and a narrow range of frequency variation (Figure 6A) [57], [59]. Similarly, p53 pulses also have a non-zero lower bound for their frequency and a narrow range of frequency variation for different strengths of γ-irradiation in the model (Figure 6A). When an oscillating current, as opposed to a constant current, with varying frequency and low amplitude is injected, type II neurons also show an interesting response. They respond sensitively to the injected current with a specific frequency [57], [59] (Figure 6B). We tested whether p53 pulses would respond in a similar way to low amplitude γ-irradiation with varying frequency, 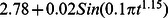 Gy. Surprisingly, p53 levels showed a sensitive response to irradiation with a specific frequency (∼0.16/hr) similar to type II neurons (Figure 6B). Mathematically type II neurons are characterized by a Hopf bifurcation when the level of external current is varied. Indeed, our p53 model also shows a supercritical Hopf bifurcation when the strength of γ-irradiation is changed.

### A Potential Coupling Mechanism of p53 Pulses among Neighboring Cells

We have shown that both p53 pulses in DNA-damaged cells and spikes of type II neurons have the characteristics of resonators. This raises the question of why DNA-damaged cells act as resonators or, in other words, what is the benefit of behaving as a resonator when p53 pulses are generated. One of the distinct characteristics of resonator neurons is that they can synchronize rhythms in purely excitatory networks, while integrator neurons cannot synchronize their rhythms [60]. This feature of the resonator neuron leads to the hypothesis that like resonator neurons, DNA-damaged cells are designed to synchronize p53 pulses.

**Figure 7:**
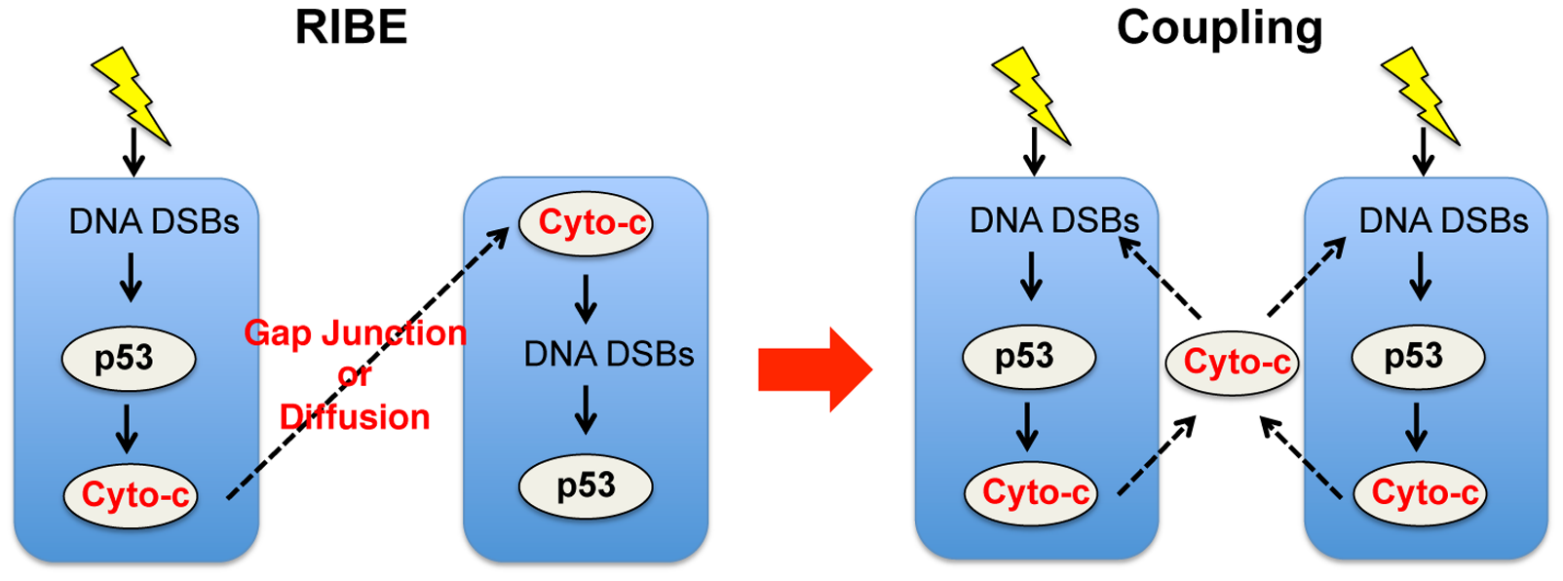
Radiation-induced bystander effects (RIBE) can be a potential mechanism that couples p53 pulses. γ-irradiation stimulates the p53-dependent release of cytochrome-c. The released cytochrome-c can stimulate the upper signal of p53 pathway of neighboring cells. This can be a potential excitatory coupling mechanism of p53 pulses among neighboring cells in response to γ-irradiation.

Before exploring this hypothesis, we first examined whether there exists a coupling signal among cells similar to excitatory neurotransmitters among neurons. Interestingly, recent studies have found that neighboring cells communicate with each other after γ-irradiation through the ‘radiation induced bystander effect (RIBE)’ [39]. RIBE is characterized by DNA damage in *un-irradiated* cells that is induced by molecular signals produced by their neighboring *irradiated* cells. While many molecular signals involving RIBE have been proposed, a recent study identified Cytochrome-c (cyt-c) as one of main signals inducing RIBE. Specifically, γ-irradiation induces the p53 dependent release of mitochondrial cyt-c, which enters *un-irradiated* neighboring cells through gap junctions [61] and diffusion [40] (Figure 7A). The released cytochrome-c then causes DSBs or DNA damage that activates p53 in the *un-irradiated* neighboring cells (Figure 7A) [62]. In this way, cyt-c can act as an excitatory neurotransmitter and provide a potential mechanism that couples p53 pulses of neighboring cells (Figure 7B). Similar couplings via diffusion of molecular signals have been identified in other biological oscillators, including the coupling of circadian rhythms of clock gene expressions via diffusion of VIP signal [63], [64] and coupling of *Dictostelium* cAMP oscillations via the diffusion of cAMP signal [29].

Given that previous studies of RIBE have explored only the effect on *un-irradiated* cells neighboring *irradiated* cells, the question remains whether the RIBE can activate p53 even in *irradiated* cells. Regarding this question, a recent study has shown the promising result that DSBs induced by RIBE, persist for a longer period than those induced by direct irradiation [65]. This suggests that RIBE has a distinct pathway for the activation of p53 different from that of direct γ-irradiation. Thus, DSBs induced by cyt-c could activate p53 even in *irradiated* neighboring cells and provide a potential coupling mechanism, although the strength of coupling may be weak (Figure 7B).

### Coupling can Synchronize p53 Pulses and Enhance the Sustainability of p53 Pulses

**Figure 8:**
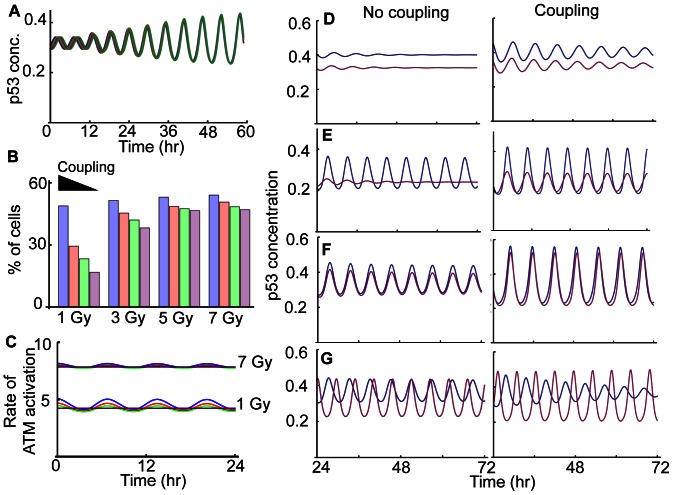
Coupling enhances the sustainability of p53 pulses. **A**. After 3 Gy irradiation, coupling through cyt-c synchronizes p53 pulses with different initial phases of four cells. **B**. As the strength of coupling becomes stronger, the fraction of cells that can sustain p53 pulses increases. Here, a heterogeneous mixture of 4000 cells is considered, with coupling of four randomly selected cells. p53 pulses were simulated for 72 hours after γ-irradiation with different strengths (1, 3, 5 and 7 Gy). For the different strength of coupling, 6, 3, 1.5 and 0 were used as β_cf,_ a coupling strength parameter (Table S3). **C**. The effect of the cyt-c signal on ATM activation becomes weaker as the strength of γ-irradiation becomes stronger. Here, β_cf_ = 6 (blue), 3 (red), 1.5 (green) and 0 (purple). **D–G.** Comparison of p53 pulses in two neighboring cells without coupling and with coupling in the presence of 1 Gy irradiation. Here, two neighboring cells were considered.

To determine whether coupling via cyt-c can induce synchronization of p53 pulses among irradiated cells, we extended the current model to include cyt-c (See Table S3 and Methods and Materials for details). In the model, cyt-c is produced in proportion to activated p53 in each cell after γ-irradiation. Total cyt-c from all neighboring cells, representing the exogenous concentration of cyt-c, then activates ATM in all neighboring cells. Here, we assumed that cyt-c, released from each cell by 3 Gy irradiation, induces DSBs of neighboring cells similar to those induced by ∼0.5 Gy irradiation, matching experimental data [40]. Although DSBs induced by cyt-c were significantly less than those induced by 3 Gy γ-irradiation, the coupling via cyt-c can synchronize p53 pulses of four neighboring cells, all of which initially had different phases (Figure 8A). Here, we considered four cells since coupling through diffusion or gap junctions would work only locally.

This result leads to the question of what might be the potential benefit of synchronization through the coupling. Because previous studies have found that synchronization through a coupling can enhance rhythm occurrence in circadian clocks and *Dictostelium* cAMP oscillators [27], , we tested whether the coupling can help to sustain p53 pulses. The sustainability of p53 pulses for 3 days in 4000 heterogeneous cells was simulated in response to different strengths of γ-irradiation. Here, parameters of every cell were perturbed by multiplying random numbers *e^X^* (*X ∼ N*(0, 0.2)) as we did previously. To test the role of coupling, we randomly coupled every four cells in the 4000 cells. Simulations showed a clear role of the coupling in sustaining p53 pulses after γ-irradiation. That is, as the strength of coupling becomes stronger, more cells can maintain their p53 pulses (Figure 8B). For instance, while two cells are unable to generate sustained p53 pulses, coupling can restore their pulses (Figure 8D). This is also seen when only one of two cells oscillates (Figure 8E). Moreover, when both cells can sustain p53 pulses with a similar period, coupling increases the amplitude of rhythms (Figure 8F). In this way, coupling enhances the occurrence of p53 rhythms, but the effect of coupling is reduced as the strength of γ-irradiation increases (Figure 8B). This is because ATM activation induced by coupling is too small to compete with activation induced by strong γ-irradiation (Figure 8C). Moreover, when both cells sustain p53 pulses with significantly different periods, coupling cannot synchronize p53 pulses and increase the amplitude, but instead coupling reduces the period difference (Figure 8G), indicating that the coupling could also play a role in tight regulation of the periods of p53 pulses. Taken together, the coupling of cells via cyt-c can synchronize p53 pulses and enhance the sustainability of the pulses. The effect of coupling becomes even more remarkable when the strength of γ-irradiation becomes weaker and the heterogeneity of periods is smaller.

## Discussion

To explore potential mechanisms that sustain p53 pulses in response to γ-irradiation, we developed a new mathematical model by modifying and extending Batchelor’s model [5]. While most mathematical models of p53 pulses are based on a single negative feedback loop between p53 and Mdm2 [11], [12], [13], [14], [15], [16], recent experimental studies have found that additional negative feedback loops are required to generate p53 pulses. While Batchelor’s model includes these newly identified negative feedback loops, it also includes explicit time delays (∼1 hr), which might be biologically unrealistic and often induce oscillation in systems whose structures are not likely to produce rhythms naturally [25], [41]. Thus, rather than including the explicit time delays, we included the intermediate steps associated with Wip1 and Mdm2 production (Figure 1A). During this process, we found that a network structure consisting of three interlocked negative feedback loops, could not sustain p53 pulses over a wide range of conditions (Figure 1B and 1D). Interestingly, when we added a recently identified positive feedback loop between p53 and Rorα, both sustainability and amplitude of the p53 pulses were significantly improved, which is consistent with previous studies showing the potential role of additional positive feedback loops [14], [22], [25] (Figure 1D and 1E). Furthermore, our new model with the additional positive feedback loop was able to reproduce many key features of p53 pulses (Figure 3–5). Our model proposes a new network structure, which can generate more robust p53 pulses: three interlocked negative feedback loops with an additional positive feedback loop. Moreover, when we included noise through stochastic simulations, we found that noise can often prevent p53 pulses from dampening even after mutations of essential species of p53 oscillatory systems, such as Wip1^−/−^, SNP309, and Rorα^−/−^ (Figure 5D–F), although the overall chances of sustained p53 pulse occurrence are significantly reduced (Figure 5G). Finally, we found that when p53 pulses in neighboring cells are coupled via cyt-c signals (Figure 7), p53 pulses are synchronized and their sustainability is enhanced unless the difference of periods were significant (Figure 8). The coupling effect becomes more significant as the strength of γ-irradiation becomes weaker (Figure 8B). Interestingly, a similar influence of coupling has been found in other biological oscillatory systems, such as circadian clocks and cAMP oscillators [27], [28], [29]. In summary, we found three potential mechanisms that enhance the sustainability of p53 pulses: 1) an additional positive feedback loop between p53 and Rorα, 2) intrinsic noise, and 3) the intercellular coupling through cyt-c.

Biological oscillatory systems can be categorized according to the driving sources of their rhythms. One category consists of endogenous oscillators (e.g. circadian clocks and sino-atrial node), which can generate rhythms without external stimuli [67], [68]. The p53 regulatory system belongs to the other category, exogenous oscillators, which require external stimuli to sustain oscillations. One of the most widely studied exogenous oscillators in cell biology is the neuron. Depending on their responses to external stimuli, most neurons can succinctly be classified as type I or type II neurons [57], [58], [59]. Type I neurons behave like an integrator, which accumulates various external current inputs that generate rhythms. Type II neurons behave like a resonator, which generate rhythms when an external current with a specific frequency is applied. We found that DNA-damaged cells behave like type II neurons (Figure 6). The fact that type II neurons easily synchronize their spikes when they are coupled through excitatory signals [60] led to the question of whether neighboring cells also have a coupling mechanism that synchronizes p53 pulses. Indeed, a recent experimental study found a potential coupling signal (cyt-c) for p53 pulses among neighboring cells (Figure 7) [40]. When we included this intercellular coupling in the model, we found that coupling through cyt-c can synchronize p53 pulses unless the difference in the periods of coupled cells is significant (Figure 8). Moreover, the coupling significantly enhances the sustainability of p53 pulses (Figure 8B).

Regarding the synchronization of p53 pulses, a high correlation between the distance among cells and their phase relationship would be an indicator of the presence of local coupling that synchronizes p53 pulses of neighboring cells. Unfortunately, most previous studies measured only the time courses of p53 pulses in individual cells without keeping track of spatial information [4], [5], [7]. We did, however, find one set of experimental data that recorded the time courses of p53 pulses among five neighboring cells [7]. Interestingly, when we analyzed this data, we found that the five cells could be categorized into two groups, each with the same peak timing of p53 pulses. This indicates that closer cells may have more similar phases or that they may synchronize p53 pulses. However, to derive a significant conclusion about the relationship between p53 phases and distance among cells, a data set much larger than five cells is required. Another interesting experiment would be to test whether cyt-c can act as a coupling mechanism of p53 pulses. For this, we first need to study whether cyt-c increases DNA damage even in *irradiated* neighboring cells since cyt-c induced DNA damage has been studied in only *un-irradiated* neighboring cells (Figure 7). If cyt-c could induce DNA damage even in *irradiated* neighboring cells, the next step would be to test whether cyt-c can synchronize p53 pulses and enhance their sustainability. The direct test for this would involve studying the effect of the inhibition of cyt-c via cyclosporine A on the p53 pulses. More specifically, future research could test whether the inhibition of cyt-c yields a wider distribution of phases and periods of p53 pulses and lower chance of sustained p53 pulses occurrence. Moreover, additional future modeling could consider a spatio-temporal modeling approach to study the role of cyt-c in depth [69].

We also found that Rorα may be an essential component in sustaining γ-triggered p53 pulses. That is, sustainability and amplitudes of p53 pulses are significantly reduced in Rorα^−/−^ cells (Figure 1D–E and 5G–H), although noise can often induce sustained p53 pulses even with Rorα^−/−^ (Figure 5F). It would be a worthwhile future experiment to test the role of Rorα on γ-triggered p53 pulses by knocking out Rorα. Interestingly, Rorα is one of core circadian (∼24 hr) clock genes, whose gene expression shows 24 hr periodic rhythms [70]. Since p53 also exhibits circadian rhythms at both the mRNA and protein level, various candidate pathways underlying p53 circadian rhythms have been proposed, such as c-myc [71], [72]. Our study proposed another potential mechanism that generates the circadian rhythms of p53 protein: positive feedback between p53 and Rorα, which could be an important target for chronotherapy. This hypothesis can be tested by considering the effect of Rorα^−/−^ on circadian rhythms of p53. A previous study suggested that noise in protein production rate with a slow correlation time (10∼20 h) could be a reason for the variability observed in γ-triggered p53 pulses [7]. Circadian rhythms of Rorα gene expression or p53 gene expression could be the source of the slowly varying noise in the protein production rates, causing a large variability in the amplitude of p53 pulses.

## Materials and Methods

### Modifications and Extensions of Model

Here, we describe new aspects of our mathematical model. The complete system of ODEs is provided in the Text S1.

#### (1) Intermediate steps instead of explicit time delays

We include intermediate steps (mRNA) for Wip1 and Mdm2 instead of explicit time delays that have been previously used in the production rates of WIP1 protein and MDM2 protein (Figure 1A and 1B). The two new ODEs that describe dynamics of Mdm2 and Wip1 mRNA are given below.
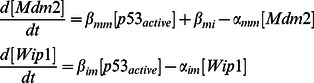



#### (2) Saturating production rate for active ATM in response to γ-irradiation

To match experimental data [47], [48], we modified production rate of active ATM so that it is more sensitive to a weak IR and saturates for strong IR (Figure 1G).
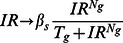



#### (3) Additional positive feedback between p53 and Rorα

A recently found positive feedback between p53 and Rorα is included in our model (Figure 1C). This adds two new ODEs to describe dynamics of Rorα mRNA and Rorα protein.
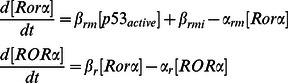



Moreover, since the MDM2-dependent degradation rate for p53 is inhibited by Rorα, the MDM2-dependent degradation rate of p53 is modeled as follows:
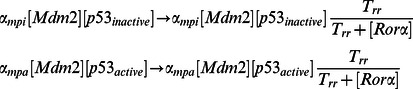



#### (4) Coupling via cytochrome-c

To include cyt-c, one ODE is added per cell (Figure 7). That is, in each cell, active p53 promotes production of cyt-c and cyt-c is degraded at rate proportional to its own concentration.
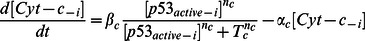



Here, sub-index *i* represents the *i*_th_ cell. To describe, cyt-c induced ATM activation, the production rate of active ATM is given by:
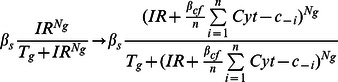



Here, the average of cyt-c produced in each cell represent exogenous concentration of cyt-c.

### Oscillation and Period Detection

To determine whether stochastically simulated time courses of p53 or Mdm2 are oscillating (Figure 3 and 5), we used autocorrelation method, which is used to detect pitch in speech. This method is also used in the analysis of the experimental data, which we used to compare with our simulations (Figure 3B) [7]. The stochastically simulated time courses are sampled every 0.05 hr and then sampled signals are smoothed through Gaussian smoothing. The autocorrelation of signals are then calculated and if the autocorrelation value is higher than 0.2, we concluded that the time courses are oscillating. Moreover, the detected pitch period is used as the period of time courses. To determine whether pulses of p53 are sustained in deterministic simulations (Figure 1 and 8), we simulated 500 hrs (∼100 cycles) in the presence of DNA damage and tested for amplitude damping.

### Parameter Search

In Figure 1D and E, parameters are randomly drawn from the uniform distributions of parameters similar to a previous study [24]. The ranges or supports of uniform distributions are described in Table S4.

### Model Simulation

All the deterministic and stochastic simulations were done with 150×8 Ghz CPU using MATHEMATICA 8.0 (Wolfram Research).

## Supporting Information

Figure S1 **Parameters with relative sensitivity higher than 0.1.** If there exists a dominant feedback loop in the biological oscillatory system, most parameters with high sensitivities are related to the core feedback loop [1], [2]. However, in our p53 model, parameters with sensitivity higher than 0.1 are associated with all species, indicating that all species and feedback loops in the model play important roles in regulating p53 pulses. Furthermore, the period of p53 pulses shows the most sensitive response to the perturbations of degradation rates of Wip1 mRNA and protein, which propose interesting future experiments.
(TIF)

Table S1
**Deterministic model parameters.** Cs = simulated concentration units. Newly added parameters are highlighted in bold. The name of parameters follows the original model [3], [4]. Sensitivity was calculated by 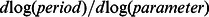 in response to 5 Gy irradiation. Minimum and maximum factor of parameters between 0 and 10 that can generate sustained p53 pulses were also calculated.(DOCX)

Table S2
**Reaction steps and probabilities of reactions in stochastic simulations.** The parameter 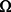 represents the number of molecules in the system. Here, we assumed that 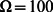 as did in previous studies [5], [6].(PDF)

Table S3 **Parameters that describe coupling through Cytochrome-C.**
(DOCX)

Table S4 **Ranges of the random parameter sets.** The random parameters were drawn from the uniform distributions in Figure 1D–E. The wider supports or ranges of uniform distributions were used for production rates than degradation rates.
(DOCX)

Text S1 **Supplementary information.**
(DOCX)
