# Supplementary figures and images for: Mechanisms That Enhance Sustainability of p53 Pulses
Source: PLoS One. 2013 Jun 3;8(6):e65242. doi: 10.1371/journal.pone.0065242 (PMC3670918; doi:10.1371/journal.pone.0065242)

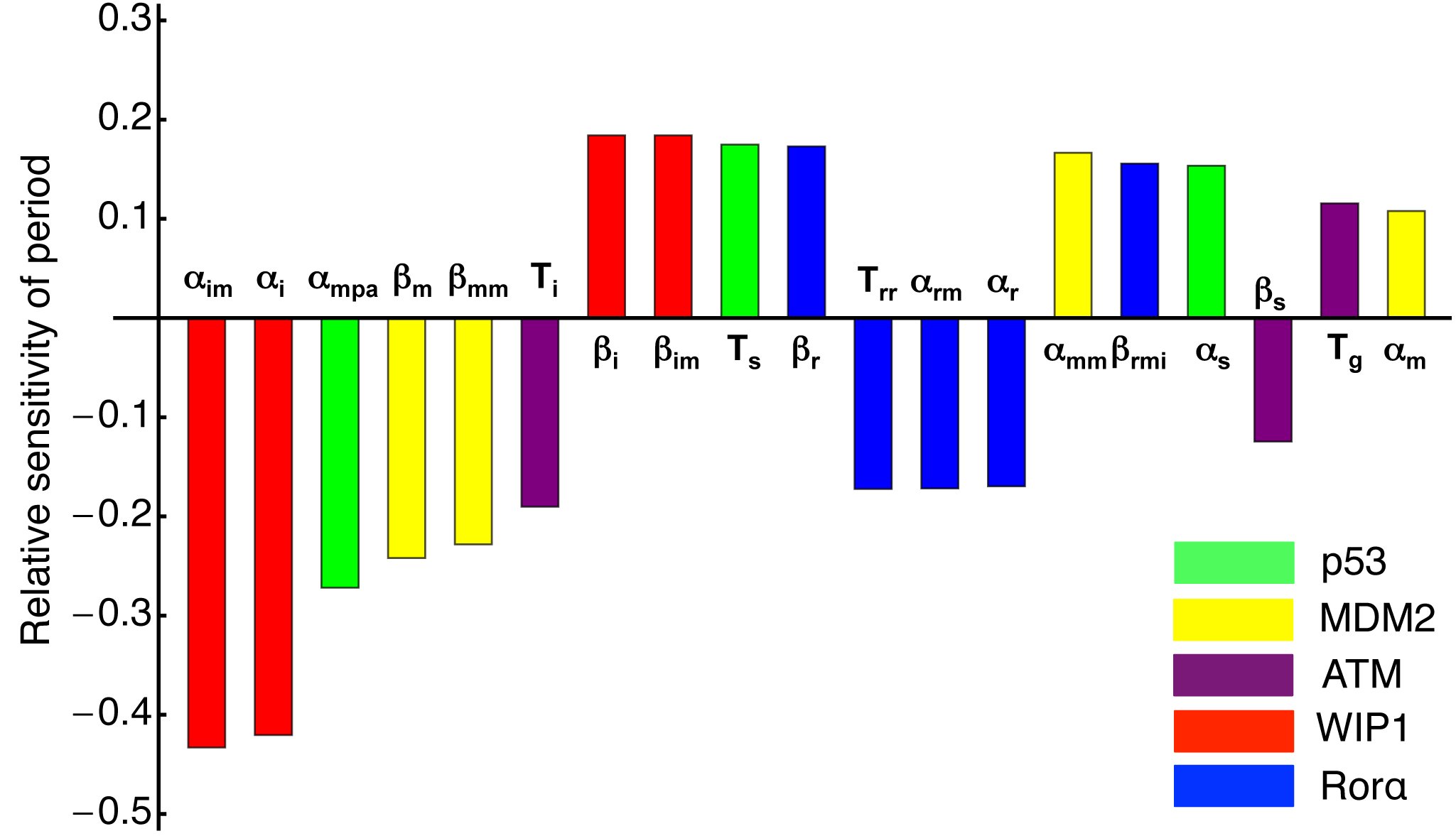

Supplement: Figure S1 — Parameters with relative sensitivity higher than 0.1. If there exists a dominant feedback loop in the biological oscillatory system, most parameters with high sensitivities are related to the core feedback loop [1], [2]. However, in our p53 model, parameters with sensitivity higher than 0.1 are associated with all species, indicating that all species and feedback loops in the model play important roles in regulating p53 pulses. Furthermore, the period of p53 pulses shows the most sensitive response to the perturbations of degradation rates of Wip1 mRNA and protein, which propose interesting future experiments. (TIF) [file pone.0065242.s001.tif]
