# Supplementary material for: Mechanisms That Enhance Sustainability of p53 Pulses
Source: PLoS One. 2013 Jun 3;8(6):e65242. doi: 10.1371/journal.pone.0065242 (PMC3670918; doi:10.1371/journal.pone.0065242)
Supplement: Table S1 — Deterministic model parameters. Cs = simulated concentration units. Newly added parameters are highlighted in bold. The name of parameters follows the original model [3], [4]. Sensitivity was calculated by in response to 5 Gy irradiation. Minimum and maximum factor of parameters between 0 and 10 that can generate sustained p53 pulses were also calculated. (DOCX) [file pone.0065242.s002.docx]

| **Name** | **Description** | **Value** | **Sensitivity** | **Min** | **Max** |
| --- | --- | --- | --- | --- | --- |
| β _p_ | p53 _inactive_ production rate | 0.9 Cs h^-1^ | -0.03 | 0.67 | >10 |
| β _sp_ | Saturating production rate of p53 _active_ | 10 h^-1^ | -0.04 | 0.44 | >10 |
| **β _mm_** | **p53-dependent Mdm2 mRNA production rate** | **9.425 h^-1^** | **-0.23** | **0.39** | **1.55** |
| **β _mi_** | **p53-independent Mdm2 mRNA production rate** | **0.08 C_s_ h^-1^** | **-0.01** | **0** | **5.71** |
| β _m_ | Mdm2 translation rate | 0.9 h^-1^ | -0.24 | 0.41 | 1.5 |
| **β _im_** | **p53-dependent Wip1 mRNA production rate** | **2.2437 h^-1^** | **0.18** | **0.79** | **3.22** |
| β _i_ | Wip1 translation rate | 0.25 h^-1^ | 0.18 | 0.79 | 3.22 |
| **β _rm_** | **p53-dependent Rorα mRNA production rate** | **0.52 h^-1^** | **0.02** | **0** | **>10** |
| **β _rmi_** | **p53-independent Rorα mRNA production rate** | **0.574 C_s_ h^-1^** | **0.16** | **0.5** | **3.3** |
| **β _r_** | **Rorα translation rate** | **1.223 h^-1^** | **0.17** | **0.53** | **2.94** |
| **β _s_** | **Saturating production rate of ATM-P** | **100 Cs h^-1^** | **-0.12** | **0.77** | **1.92** |
| α _mpi_ | Saturating MDM2 and Rorα dependent p53_inactive_ degradation rate | 5 Cs^-1^ h^-1^ | 0.03 | 0 | 4.94 |
| α _pi_ | p53_inactive_ degradation rate | 2 h^-1^ | 0.02 | 0 | 2.46 |
| α _ipa_ | Wip1-dependent p53_active_ inactivation rate | 0.14 Cs^-1^ h^-1^ | 0.01 | 0 | >10 |
| α _mpa_ | Saturating Mdm2 and Rorα dependent p53_active_ degradation rate | 1.4 Cs^-1^ h^-1^ | -0.27 | 0.39 | 1.5 |
| **α _mm_** | **Mdm2 mRNA degradation rate** | **0.583 h^-1^** | **0.17** | **0.69** | **1.87** |
| α _m_ | MDM2 degradation rate | 1 h^-1^ | 0.11 | 0.47 | 1.77 |
| α _sm_ | ATM-P-dependent MDM2 inactivation rate | 0.5 Cs^-1^  h^-1^ | 0.05 | 0.15 | >10 |
| **α _im_** | **Wip1 mRNA degradation rate** | **0.769 h^-1^** | **-0.43** | **0.43** | **1.25** |
| α _i_ | WIP1 degradation rate | 0.7 h^-1^ | -0.42 | 0.48 | 1.29 |
| **α _rm_** | **Rorα mRNA degradation rate** | **0.295 h^-1^** | **-0.17** | **0.33** | **1.86** |
| **α _r_** | **RORα degradation rate** | **1.091 h^-1^** | **-0.17** | **0.33** | **1.91** |
| α _is_ | Saturating WIP1-dependent ATM-P degradation rate | 50 h^-1^ | 0.06 | 0.73 | >10 |
| α _s_ | WIP1-independent ATM-P degradation rate | 7.5 h^-1^ | 0.01 | 0 | 1.16 |
| T_s_ | ATM-P concentration for half-maximal p53 production | 1 Cs | 0.17 | 0.57 | 1.34 |
| T_i_ | WIP1 concentration for half-maximal ATM-P degradation | 0.2 Cs | -0.19 | 0.32 | 1.26 |
| **T_rr_** | **RORα concentration for half-maximal p53 degradation** | **0.976 Cs^-1^** | **-0.17** | **0.34** | **1.89** |
| **T_g_** | **DNA damage for half-maximal ATP-production** | **24** | **0.12** | **0.48** | **1.31** |
| n_s_ | Hill coefficient of active p53 production by ATM-P | 4 | 0.15 | 0.88 | >10 |
| n_i_ | Hill coefficient of ATM-P degradation by WIP1 | 4 | -0.03 | 0.88 | >10 |
| **IR** | **Strength of γ-irradiation** | **0 ~10 Gy** | **-0.01** | **0.51** | **>10** |
| **n_g_** | **Constant converting strength of γ-irradiation to DNA damage** | **0.4** | **-0.07** | **0.58** | **2.13** |
|  | p53 _inactive_ initial condition | 0.243 Cs |  |  |  |
|  | p53 _active_ initial condition | 0.077 Cs |  |  |  |
|  | Mdm2 mRNA initial condition | 1.065 Cs |  |  |  |
|  | MDM2 initial condition | 2.336 Cs |  |  |  |
|  | Wip1 mRNA initial condition | 0.081 Cs |  |  |  |
|  | WIP1 initial condition | 0.348 Cs |  |  |  |
|  | Rorα mRNA initial condition | 1.39 Cs |  |  |  |
|  | RORα initial condition | 0.226 Cs |  |  |  |
|  | ATM-p initial condition | 2.083 Cs |  |  |  |
