# Supplementary material for: Mechanisms That Enhance Sustainability of p53 Pulses
Source: PLoS One. 2013 Jun 3;8(6):e65242. doi: 10.1371/journal.pone.0065242 (PMC3670918; doi:10.1371/journal.pone.0065242)
Supplement: Table S2 — Reaction steps and probabilities of reactions in stochastic simulations. The parameter represents the number of molecules in the system. Here, we assumed that as did in previous studies [5], [6]. (PDF) [file pone.0065242.s003.pdf]

Reaction number	Reaction step	Probability of Reaction
1	$* \rightarrow p53_{inactive}$	$\beta_P \times \Omega$
2	$p53_{inactive} \rightarrow *$	$\alpha_{mpi} \times [p53_{inactive}] \times [MDM2] \times \frac{\Omega T_{rr}}{\Omega T_{rr} + [ROR\alpha]}$
3	$p53_{inactive} \rightarrow *$	$\alpha_{pi} \times [p53_{inactive}]$
4	$p53_{inactive} \rightarrow p53_{active}$	$\beta_{sp} \times [p53_{inactive}] \times \frac{[ATM]^{n_s}}{(T_s \times \Omega)^{n_s} + [ATM]^{n_s}}$
5	$p53_{active} \rightarrow *$	$\alpha_{mpa} \times [p53_{active}] \times [MDM2] \times \frac{\Omega T_{rr}}{\Omega T_{rr} + [ROR\alpha]}$
6	$p53_{active} \rightarrow p53_{inactive}$	$\alpha_{ipa} \times [p53_{active}] \times [WIP1]$
7	$* \rightarrow Mdm2$	$\beta_{mi} \times \Omega$
8	$* \rightarrow Mdm2$	$\beta_{mm} \times [p53_{active}]$
9	$Mdm2 \rightarrow *$	$\alpha_{mm} \times [Mdm2]$
10	$* \rightarrow MDM2$	$\beta_m \times [Mdm2]$
11	$MDM2 \rightarrow *$	$\alpha_m \times [MDM2]$
12	$MDM2 \rightarrow *$	$\alpha_{sm} \times [MDM2] \times [ATM] / \Omega$
13	$* \rightarrow Ror\alpha$	$\beta_{rmi} \times \Omega$
14	$* \rightarrow Ror\alpha$	$\beta_{rm} \times [p53_{active}]$
15	$Ror\alpha \rightarrow *$	$\alpha_{rm} \times [Ror\alpha]$
16	$* \rightarrow ROR\alpha$	$\beta_r \times [Ror\alpha]$
17	$ROR\alpha \rightarrow *$	$\alpha_r \times [ROR\alpha]$
18	$* \rightarrow Wip1$	$\beta_{im} \times [p53_{active}]$
19	$Wip1 \rightarrow *$	$\alpha_{im} \times [Wip1]$
20	$* \rightarrow WIP1$	$\beta_i \times [Wip1]$
21	$WIP1 \rightarrow *$	$\alpha_i \times [WIP1]$
22	$* \rightarrow ATM$	$\beta_s \times \Omega \times \frac{IR^{n_g}}{T_g + IR^{n_g}}$

23	$ATM \rightarrow *$	$\alpha_{is} \times [ATM] \times \frac{[WIP1]^{n_i}}{(T_i \times \Omega)^{n_i} + [WIP1]^{n_i}}$
24	$ATM \rightarrow *$	$\alpha_i \times [ATM]$
