# Supplementary material for: Mechanisms That Enhance Sustainability of p53 Pulses
Source: PLoS One. 2013 Jun 3;8(6):e65242. doi: 10.1371/journal.pone.0065242 (PMC3670918; doi:10.1371/journal.pone.0065242)
Supplement: Table S3 — Parameters that describe coupling through Cytochrome-C. (DOCX) [file pone.0065242.s004.docx]

| **Parameter** | **Description** | **Value** |
| --- | --- | --- |
| β _c_ | Saturating p53-dependent Cyt-c production rate | 1 h^-1^ |
| α _c_ | Cyt-c degradation rate | 5 h^-1^ |
| n_c_ | Hill coefficient of Cyt-c production by p53_active_ | 2 |
| β _cf_ | Strength of coupling | 6 |
| T _c_ | p53 concentration for half-maximal Cyt-c production | 0.1 |
