# Supplementary material for: Mechanisms That Enhance Sustainability of p53 Pulses
Source: PLoS One. 2013 Jun 3;8(6):e65242. doi: 10.1371/journal.pone.0065242 (PMC3670918; doi:10.1371/journal.pone.0065242)
Supplement: Table S4 — Ranges of the random parameter sets. The random parameters were drawn from the uniform distributions in Figure 1D–E. The wider supports or ranges of uniform distributions were used for production rates than degradation rates. (DOCX) [file pone.0065242.s005.docx]

| **Name** | **Description** | **Range** |
| --- | --- | --- |
| β _mm_ | p53-dependent Mdm2 mRNA production rate | 0-50 |
| β _mi_ | p53-independent Mdm2 mRNA production rate | 0-50 |
| β _im_ | p53-dependent Wip1 mRNA production rate | 0-50 |
| β _rm_ | p53-dependent Rorα mRNA production rate | 0-50 |
| β _rmi_ | p53-independent Rorα mRNA production rate | 0-50 |
| β _r_ | Rorα translation rate | 0-50 |
| α _mm_ | Mdm2 mRNA degradation rate | 0-10 |
| α _im_ | Wip1 mRNA degradation rate | 0-10 |
| α _rm_ | Rorα mRNA degradation rate | 0-10 |
| α _r_ | RORα degradation rate | 0-10 |
| T_rr_ | RORα concentration for half-maximal p53 degradation | 0-10 |
