# Supplementary material for: Mechanisms That Enhance Sustainability of p53 Pulses
Source: PLoS One. 2013 Jun 3;8(6):e65242. doi: 10.1371/journal.pone.0065242 (PMC3670918; doi:10.1371/journal.pone.0065242)
Supplement: Text S1 — Supplementary information. (DOCX) [file pone.0065242.s006.docx]

**Supplementary Information**

**Mechanisms that enhance sustainability of p53 pulses**

Jae Kyoung Kim^1^ and Trachette L. Jackson^1*^

1 Department of Mathematics, University of Michigan, Ann Arbor, Michigan 48109, USA

* * To whom correspondence should be addressed: TLJ (tjacks@umich.edu)

**Equations**

**Ordinary Differential Equations for the p53 Model**

**Ordinary Differential Equations for the Coupling**

$$\begin{matrix} \frac{d[Cyt-c_{-i}]}{dt}=\beta_{c}\frac{[p53_{active-i}]^{n_{c}}}{[p53_{active-i}]^{n_{c}}+T_{c}^{n_{c}}}-\alpha{}_{c}[Cyt-c_{-i}] \\ \frac{d[ATM]}{dt}=\beta_{s}\frac{(IR+\frac{\beta_{cf}}{n}\sum_{i=1}^{n} Cyt-c_{-i})^{n_{g}}}{T_{g}+(IR+\frac{\beta_{cf}}{n}\sum_{i=1}^{n} Cyt-c_{-i})^{n_{g}}}-\alpha_{is}\frac{[WIP1]^{n_{i}}}{[WIP1]^{n_{i}}+T_{i}^{n_{i}}}[ATM]-\alpha_{s}[ATM] \end{matrix}$$

**Supporting Information Legend**

**Figure S1. Parameters with relative sensitivity higher than 0.1.** If there exists a dominant feedback loop in the biological oscillatory system, most parameters with high sensitivities are related to the core feedback loop [[1](#_ENREF_1),[2](#_ENREF_2)]. However, in our p53 model, parameters with sensitivity higher than 0.1 are associated with all species, indicating that all species and feedback loops in the model play important roles in regulating p53 pulses. Furthermore, the period of p53 pulses shows the most sensitive response to the perturbations of degradation rates of Wip1 mRNA and protein, which propose interesting future experiments.

**Table S1. Deterministic model parameters**. Cs = simulated concentration units. Newly added parameters are highlighted in bold. The name of parameters follows the original model [[3](#_ENREF_3),[4](#_ENREF_4)]. Sensitivity was calculated by $d\log(period)/d\log(parameter)$ in response to 5Gy irradiation. Minimum and maximum factor of parameters between 0 and 10 that can generate sustained p53 pulses were also calculated.

**Table S2. Reaction steps and probabilities of reactions in stochastic simulations.** The parameter $\Omega$ represents the number of molecules in the system. Here, we assumed that $\Omega=100$ as did in previous studies [[5](#_ENREF_5),[6](#_ENREF_6)].

**Table S3. Parameters that describe coupling through Cytochrome-C.**

**Table S4. Ranges of the random parameter sets**. The random parameters were drawn from the uniform distributions in Figure 1D-E. The wider supports or ranges of uniform distributions were used for production rates than degradation rates.

**Supplementary Reference**

1. Kim JK, Forger DB (2012) A mechanism for robust circadian timekeeping via stoichiometric balance. Molecular systems biology 8: 630.

2. Wilkins AK, Barton PI, Tidor B (2007) The Per2 negative feedback loop sets the period in the mammalian circadian clock mechanism. PLoS computational biology 3: e242.

3. Batchelor E, Loewer A, Mock C, Lahav G (2011) Stimulus-dependent dynamics of p53 in single cells. Molecular systems biology 7: 488.

4. Batchelor E, Mock CS, Bhan I, Loewer A, Lahav G (2008) Recurrent initiation: a mechanism for triggering p53 pulses in response to DNA damage. Molecular cell 30: 277-289.

5. Ouattara DA, Abou-Jaoude W, Kaufman M (2010) From structure to dynamics: frequency tuning in the p53-Mdm2 network. II Differential and stochastic approaches. Journal of theoretical biology 264: 1177-1189.

6. Gonze D, Halloy J, Goldbeter A (2002) Deterministic versus stochastic models for circadian rhythms. Journal of Biological Physics 28: 637-653.
